# AKASI and Near-Infrared Spectroscopy in the combined effectiveness evaluation of an actinic keratoses preventive product in immunocompetent and immunocompromised patients

**DOI:** 10.3389/fmed.2022.987696

**Published:** 2022-09-07

**Authors:** Federica Veronese, Silvia Seoni, Vanessa Tarantino, Matteo Buttafava, Chiara Airoldi, Kristen M. Meiburger, Elisa Zavattaro, Paola Savoia

**Affiliations:** ^1^SCDU Dermatologia, AOU Maggiore della Carità, Novara, Italy; ^2^Biolab, PolitoBIOMed Lab, Department of Electronics and Telecommunications, Politecnico di Torino, Turin, Italy; ^3^School of Medicine, University of Eastern Piedmont, Novara, Italy; ^4^Department of Translational Medicine, University of Eastern Piedmont, Novara, Italy; ^5^Department of Health Sciences, University of Eastern Piedmont, Novara, Italy

**Keywords:** prevention, immunosuppression, actinic keratoses, AKASI, NIRS

## Abstract

**Introduction:**

The high incidence of actinic keratoses among both the elderly population and immunocompromised subjects and the considerable risk of progression from *in situ* to invasive neoplasms makes it essential to identify new prevention, treatment, and monitoring strategies.

**Objective:**

The aim of this study was to evaluate the efficacy on AKs of a topical product (^®^Rilastil AK Repair 100 +) containing high-protection sunscreens, a DNA Repair Complex with antioxidant and repairing action against UV-induced DNA damage, and nicotinamide, a water-soluble derivative of vitamin B3 that demonstrated several photoprotective effects both *in vitro* and *in vivo*.

**Methods:**

The study enrolled 74 Caucasian patients, which included 42 immunocompetent and 32 immunosuppressed subjects. The efficacy of the treatment has been evaluated through the clinical index AKASI score and the non-invasive Near-Infrared Spectroscopy method.

**Results:**

The AKASI score proved to be a valid tool to verify the efficacy of the product under study, highlighting an average percentage reduction at the end of treatment of 31.37% in immunocompetent patients and 22.76% in organ transplant recipients, in comparison to the initial values, with a statistically significant reduction also in the single time intervals (T0 vs. T1 and T1 vs. T2) in both groups. On the contrary, the Near-Infrared Spectroscopy (a non-invasive technique that evaluates hemoglobin relative concentration variations) did not find significant differences for O_2_Hb and HHb signals before and after the treatment, probably because the active ingredients of the product under study can repair the photo-induced cell damage, but do not significantly modify the vascularization of the treated areas.

**Conclusion:**

The results deriving from this study demonstrate the efficacy of the product under study, confirming the usefulness of the AKASI score in monitoring treated patients. Near-Infrared Spectroscopy could represent an interesting strategy for AK patients monitoring, even if further large-scale studies will be needed.

## Introduction

In the last decades, the concept of “field cancerization” (FC), a biological process in which large skin surfaces are affected by carcinogenic alterations, and the consequent need for treatment of actinic keratoses (AKs), has become more and more important.

Figueras-Nart et al. has recently defined the skin FC as an area affected by multiple AKs, together with visible sun damage and at least two of the following characteristics: atrophy, telangiectasias, hypo/hyperpigmented skin and “glass paper skin” (1). In this area, histologically, it is possible to recognize sub-clinical lesions with atypical keratinocytes, nuclear pleomorphism, loss of cell polarity, dermal solar elastosis and vascular ectasia (2), together with obvious AKs, that are characterized by the presence of parakeratosis and lymphocytic infiltrate (3).

Patients with FC have a higher risk of developing Squamous Cell Carcinoma (SCC) than individuals with isolated AKs (4). For this reason, it’s important to treat the whole FC since the treatment of the single lesion is associated to the risk of evolution of the visible and sub-clinical AKs of the surrounding area (5).

In literature, the annual risk of evolution in invasive SCC (iSCC) is estimated of 0.075% for a single AK in individuals without a personal history of Non-Melanoma Skin Cancer (NMSC) and 0.53% for individuals with previous lesions (6). Also, NMSCs are the most frequent cancers in immunosuppressed patients, with 27% of patients affected, and steady incidence increase over the years as the immunosuppression time progresses (7, 8).

In this context, not only the treatment but also the prevention of the development and evolution of AKs acquires a fundamental role. The use of photoprotection is recommended as an adjuvant for any type of treatment (9, 10). Daily use of sunscreen with SPF 50 + favors the spontaneous regression of AKs and reduces the incidence of iSCC in both immunocompetent and immunosuppressed patients (8, 10–12).

However, sunscreens protect against UV-induced DNA damage but are not effective once the damage has been established; for this reason, Krutmann et al. (13) report that, especially in high- and very high-risk subjects (Table 1), active ingredients capable of repairing DNA damage (e.g., endonuclease and photolyase) should be employed during the whole year.

**TABLE 1:** Patients requiring use of sunscreens and DNA repair ingredients.

High-risk	Very high-risk
Presence of Aks	Presence of > 10 Aks in the FC
Previous treatment for Aks	Immunosuppressed patients or organ transplant recipients (OTRs)
Previous NMSC in immunocompetent patients	Patients affected by Xeroderma Pigmentosus
Patients with clinical signs of skin photodamage	

Several studies have been recently conducted on devices containing sunscreens (SPF 50–100 +) and DNA repair enzymes (10, 14–16); in particular, in 2019 we carried out a study concerning the efficacy of the product ^®^Rilastil AK Repair 100 + (a photoprotector containing SPF UVB 131 and UVA 53, and a DNA Repair Complex (DRC) with antioxidant and repairing action against UV-induced DNA damage) compared to a simple photoprotector (17).

The product formulation is reported in Table 2 and has been recently modified with the introduction of nicotinamide instead of vitamin E. In this paper, we used the clinical index for AKs AKASI score and a non-invasive Near-Infrared Spectroscopy (NIRS) method to evaluate the efficacy of the new product formulation.

**TABLE 2:** ^®^Rilastil AK Repair 100 + composition.

Active principles	Function
Physical and Chemical filters UVB-UVA	Photoprotection
DNA Repair Complex (amino acids, vegetable hydrolyzed proteins, ATP)	DNA repair and antioxidant
Epigallocatechin gallate	Antioxidant
Nicotinamide	Photoprotection and anti-inflammatory

## Materials and methods

### Study design

This was a prospective observational study conducted in our Dermatology Unit, in Novara (Italy). Continuous enrollment was carried out between patients visited both in general clinics and in those specifically dedicated to transplant carriers from March to September 2021, with a follow-up extended until February 2022. The study protocol was approved by the Local Ethical Committee (CE80/18) and was conducted in accordance with the Helsinki’s Declaration.

The study population consisted of 74 Caucasian patients: 42 immunocompetent and 32 immunosuppressed, among which 31 organ transplant recipients (OTRs) and 1 under treatment with Teriflunomide. Inclusion criteria were: i) age > 40 years; ii) clinical evidence of AKs (grade I or II) on the face and scalp; iii) personal history of previous NMSC; and, only for OTRs, iv) immunosuppressive treatment for at least 5 years; exclusion criteria were represented by the incapacity to sign the informed consent and to properly apply the products or by the presence of genetic disorders conditioning the development of NMSCs (i.e., Gorlin-Goltz syndrome, Xeroderma Pigmentosums, Epidermodysplasia Verruciformis).

At the baseline visit (T0) we collected the information about patient personal data (gender, age), endogenous and exogenous risk factor (phototype, previous actinic burns, history of NMSC, occupational and/or recreational UV exposure, artificial UV exposure, use of sunscreens), and previous treatment for AKs. The participants were instructed to apply the product under study (^®^Rilastil AK Repair 100 +) to the photo-damaged areas twice daily (morning and early afternoon) for 6 months. The cream was provided by the manufacturer (Ganassini Corporate, Milano) to patients free of charge; patients were asked to return the finished tubes, to verify the application of the correct amount of the product.

The patients were re-evaluated after 3 (T1) and 6 (T2) months. At each visit, photographic documentation was collected, and the AKASI score was calculated, based on literature suggestions (18, 19). In detail, the head was divided into four regions (scalp, forehead, left/right cheek, ear, chin, and nose). In each region, the percentage of the area affected by AKs was estimated (score 0–6), and the severity of three clinical AK signs (distribution, erythema, and thickness) was assessed (score 0–4 for each parameter). The total score ranges from 0 to 18 (18).

**FIGURE 1:**
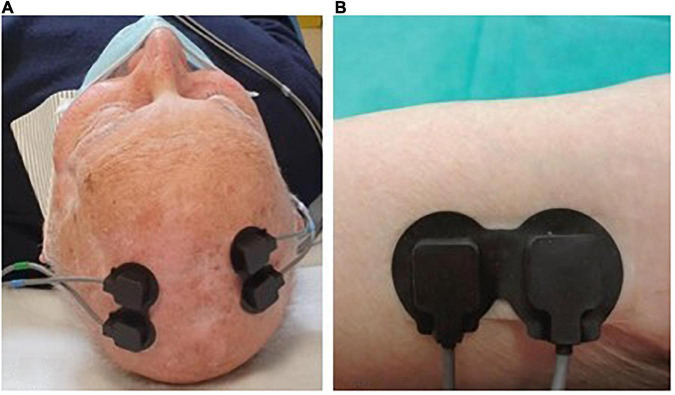
Example of positioning the probes on Aks **(A)** and on healthy skin **(B)**.

Fifteen patients (8 immunocompetent and 7 immunosuppressed) were also evaluated through NIRS spectroscopy at T0 and T2. In collaboration with PoliToBIOMed Lab (Turin Polytechnic University) we acquired the change in the relative concentration of oxygenated (O_2_Hb) and deoxygenated (HHb) hemoglobin (Hb), using the commercial device NIRO^®^ 200-NX (Hamamatsu). The signals were acquired at a sampling frequency of 5 Hz. All signals were filtered using a band-pass Chebyshev filter, in order to remove the frequencies outside the range 10–250 mHz. The instrument has two probes: the emitter (LED light source with wavelengths of 735–810 nm) and the detector (a photodiode). The distance between the two probes influences the depth of the analyzed area and the assessed area of the body. For our application, we placed the probes at a distance of 2.5 cm from each other, in order to analyze the superficial area under the skin. For each patient, signals were acquired both on healthy skin (no UV-induced damage) and on the AK site (before and after treatment) (Figure 1).

For the evaluation of the vascular response of the tissue, the NIRS signals were acquired according to the following protocol (20, 21):

- acquisition of the baseline signal for 1.5 min.
- application of an ice pack near the probes (vasoconstrictor stimulus) for 1.5 min.
- removal of the ice pack and acquisition of vascular response for 1.5 min.

This acquisition protocol allows us to verify if there is a different vascular response to a vasoconstrictor stimulus (i.e., the ice pack application) between T0 and T2.

### Statistical analysis

We performed a descriptive analysis of the whole study population and separately for immunocompetent and immunosuppressed. Absolute and relative frequencies were reported for categorical data while, for numerical one, we used mean and standard deviations (SD) or median and interquartile range, as appropriate. Demographic and clinical data between immunocompetent and immunosuppressed were compared and results of chi-square or Fisher test and *t*-test were reported. Moreover, the AKASI score at T0 was calculated for different levels of risk factor and *t*-tests were performed.

Then, we evaluated the differences of AKASI score between time (T1 vs. T0 and T2 vs. T0) separately for immunocompetent and immunosuppressed and statistical significativity was tested using Wilcoxon paired *t*-tests. We also compared the difference among the two groups of patients. Moreover, we calculated the percentage variation in time between time. Finally, the proportion of subjects who achieved a reduction of at least 50–75–100% in AKASI score at the end of the study was also evaluated.

**FIGURE 2:**
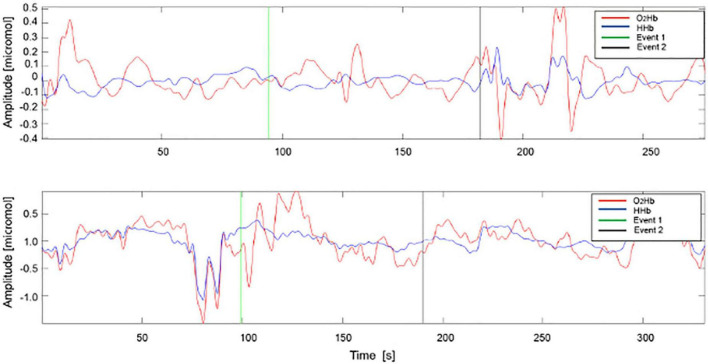
Example of acquisition of O_2_Hb and HHb signals in AK before **(A)** and after **(B)** treatment. HHb signals are represented in blue and O_2_Hb signals in red. Event 1: ice application, Event 2: ice removal.

For the NIRS signal analysis, the dataset consists of 18 signals acquired on the lesion before and after treatment with the cream. Three patients presented more than one lesion on which the NIRS analysis was done, hence increasing the NIRS dataset by 3 when compared to the number of analyzed subjects. The signals were divided into three moments of acquisition (baseline, ice application and after ice removal). The analyzed epochs are the following:

- O_2_Hb before: variation in oxygenated Hb concentration before ice application;
- O_2_Hb after: variation after ice pack application;
- HHb before: variation of the deoxygenated Hb concentration before ice application;
- HHb after: variation after ice application (see Figure 2).

During the ice pack application, the signals were often corrupted by artifacts, such as the motion artifacts, hence only the baseline and after ice removal epochs were analyzed. For all signals acquired on the lesion, pre- and post-treatment and on healthy skin, 18 parameters were estimated, 14 in the time domain and 4 in the frequency domain. The AK lesion parameters before and after-treatment were normalized with the parameters of the healthy skin, maintaining the correspondence of the patient.

A multivariate analysis of variance (MANOVA) was applied to the 18 time and frequency parameters to the verify if there were statistical differences between the vascular response before and after the treatment. The *p*-value and MANOVA dimension (d) were used to determine whether the two groups showed statistically significant differences and could be considered as belonging to two separate groups (*d* = 1). The statistical analysis was composed by 4 MANOVA tests (HHb before, HHb after ice application, O_2_Hb before and O_2_Hb after ice application), and for this we applied the Bonferroni correction to re-define the significance level α equal to 0.0125 (i.e., 0.05/4).

The clinical data collected were analyzed with SAS 9.4 software and the significance level was set at 0.05.

## Results

### Clinical characteristics of the study population

The statistical analysis was performed considering separately the immunocompetent and immunocompromised subjects. General analyses at T0 were conducted to verify any statistically significant differences between these two populations.

The clinical characteristics and risk factors of the study population, global and specific of the two subgroups, are reported in Table 3. Particularly, the mean ± SD value of AKASI score found in the total of subjects at T0 was equal to 3.92 ± 2.20; in the immunocompetent population, it was 4.47 ± 2.26, while among the immunosuppressed it was 3.21 ± 1.94.

**TABLE 3:** A descriptive comparison of risk factors and anamnestic data between the two groups of patients.

	Total patients (*n* = 74)		Immunocompetent (*n* = 42)		Immunosuppressed (*n* = 32)		*p*-value
	N	%	N	%	N	%	
**Gender**							
Female	12	16.22	9	21.43	3	9.38	0.163
Male	62	83.78	33	78.57	29	90.63	
**Age**							
Mean ± SD	73.55 ± 10,61		77.67 ± 9,30		68.16 ± 9.89		**<0.001**
**Outdoor Profession**							
Yes	27	36.49	14	33.33	13	40.63	0.518
No	47	63.51	28	66.67	19	59.37	
**Phototype**							
II	46	62.16	37	88.10	9	28.13	**<0.001**
III	28	37.84	5	11.90	23	71.88	
**Previous sun exposure**							
Intensive	41	55.41	24	57.14	17	53.13	0.735
Occasional	33	44.59	18	42.86	15	46.87	
**Use of photoprotectors**							
Yes	25	33.78	9	21.43	16	50	**0.010**
No	49	66.22	33	78.57	16	50	
**Previous actinic burns**							
Yes	35	47.30	21	50	14	43.75	0.593
No	39	52.7	21	50	18	56.25	
**Artificial UV exposure**							
Yes	5	6.76	3	7.14	2	6.25	0.879
No	69	93.24	39	92.86	30	93.75	
**AKASI score T0**							
Mean ± SD	3.92 ± 2.20		4.47 ± 2.26		3.21 ± 1.94		**0.0013**
**Previous NMSC**							
Yes	54	72.97	31	73.81	23	71.88	0.852
No	20	27.03	11	26.19	9	28.12	
**Previous NMSC type (*n* = 54)**							
SCC	14	25.93	9	29.03	5	21.74	0.7461
BCC	26	48.15	15	48.39	11	47.83	
SCC and BCC	14	25.93	7	22.58	7	30.43	
**Previous Aks treatment**							
Yes	63	85.14	36	85.71	27	84.38	0.872
No	11	14.86	6	14.29	5	15.62	
**Treatments type**							
Photodynamic therapy	3	4.05	2	4.76	1	3.13	0.723
Local therapy*	25	33.78	13	30.95	12	37,50	0.555
Surgery	37	50	18	42.86	19	59,38	0.159
Cryotherapy	54	72.97	32	76.19	22	68.75	0.475
**Use of analogs of ^®^AK Repair**							
Yes	17	22.97	8	19.05	9	28.13	0.357
No	57	77.03	34	80.95	23	71.87	

Statistically significant values are in bold. * The following therapies are included: imiquimod, 5% -FU, piroxicam, ingenol mebutate.

Previous treatment for AKs had been overall carried out by 63 (85.14%) subjects (36 immunocompetent and 27 immunosuppressed). Moreover, 17 subjects (22.97%), reported previous use of a similar product.

From this preliminary analysis, it emerged that immunocompetent patients had a significantly higher average age than immunosuppressed subjects (respectively 77.67 ys and 68.16 ys); moreover, the percentage of subjects with phototype II was significantly higher among the immunocompetent than immunosuppressed (88.10% vs. 28.13%). It should be noted that all subjects included in the study, both in the immunocompetent subgroup and in the transplant subgroup, had phototype II or III.

There was also a statistically significant difference in the two groups regarding the previous use of photoprotectors, which was higher in immunosuppressed patients (50% vs. 21.43%), with a *p*-value of 0.010.

Therefore, we analyzed all the possible associations between known risk factors for AKs and the AKASI score at T0; in addition, the association between the AKASI score at T0 and the presence or absence of previous treatments for AKs, use of analogs of the medical device under study and other NMSCs was also evaluated.

The difference in the mean AKASI score at T0 between immunosuppressed and immunocompetent patients reaches a *p*-value of 0.013 (3.21 ± 1.94 and 4.47 ± 2.26, respectively).

The 46 subjects with phototype II have an average AKASI score of 4.37 ± 2.18 at the time of enrollment, while the 28 subjects with phototype III of 3.19 ± 2.08 (*p*-value 0.024).

Finally, the 54 subjects with a previous NMSC diagnosis have an average AKASI score at T0 of 4.29 ± 2.29, and the 20 subjects without previous NMSC of 2.95 ± 1.63 (*p*-value 0.019).

Subsequently, the partial AKASI scores were analyzed in the 74 subjects at T0 and an average AKASI score on the scalp was found to be greater than the other three partial areas.

All the results are reported in Table 4.

**TABLE 4:** Association between risk factors and anamnestic data with the mean AKASI score at T0.

Variable	N	AKASI score T0 mean ± SD	AKASI score T0 median (IQR)	*p*-value
**Gender**				
Female	12	3.13 ± 1,81	3.40 (2.7)	0.176
Male	62	4.08 ± 2.25	3.60 (3.4)	
**Immunosuppression**				
Yes	32	3.21 ± 1.94	2.50 (2.8)	**0.013**
No	42	4.47 ± 2,26	4.20 (2.4)	
**Outdoor profession**				
Yes	27	4.02 ± 2.01	3.60 (3.2)	0.774
No	47	3.87 ± 2.33	3.60 (3.4)	
**Phototype**				
II	46	4.37 ± 2.18	4.10 (2.4)	**0.024**
III	28	3.19 ± 0.08	2.40 (3.3)	
**Previous sun exposure**				
Intensive	41	4.35 ± 2.43	3.80 (3.6)	0.062
Occasional	33	3.39 ± 1.78	3.20 (2.6)	
**Use of photoprotection**				
Yes	25	3.33 ± 1.84	2.60 (2.8)	0.096
No	49	4.23 ± 2.33	4 (2.8)	
**Previous actinic burns**				
Yes	35	4.34 ± 2.14	4.20 (2.8)	0.127
No	39	3.55 ± 2.22	3.40 (2.8)	
**Artificial UV exposure**				
Yes	5	4.60 ± 4.03	4 (2,4)	0.481
No	69	3.88 ± 2.06	3.60 (3.2)	
**Previous NMSC**				
Yes	54	4.29 ± 2.29	3.90 (3.2)	**0.019**
No	20	2.95 ± 1.63	2.80 (2.2)	
**Previous NMSC type (*n* = 54)**				
SCC	14	4.03 ± 2.03	3.60 (3)	0.129
BCC	26	3.85 ± 2.41	3.40 (3)	
SCC and BCC	14	5.34 ± 2.12	5.40 (2)	
**Use of analogs of Rilastil AK repair**				
Yes	17	4.31 ± 1.91	3.80 (2.4)	0.419
No	57	3.81 ± 2.29	3.40 (3.2)	
**Partial AKASI score at T0 (Mean ± SD)**				
Scalp		1.55 ± 1.63		
Forehead		0.89 ± 0.72		
Right half face		0.79 ± 0.64		
Left half face		0.70 ± 0.63		

Statistically significant values are in bold. At the bottom of the table, AKASI score averages stratified based on the location.

### Response to treatment

Overall, from the 74 patients treated with^®^Rilastil AK Repair 100 +, 6 patients between T0 and T1 and 4 patients between T1 and T2 were lost to follow-up. So, the analysis of the AKASI score trend resulting from the treatment was carried out on 68 patients (39 immunocompetent and 29 immunosuppressed) between T0 and T1 and 64 patients (37 immunocompetent and 27 immunosuppressed) between T0 and T2.

The AKASI seemed to decrease among time: at T0 the mean score was 3.92 (SD 2.20), at T1 3.16 (SD 2.05) while in the last visit it was 2.73 (SD 1.64). When we analyzed immunocompetent and immunosuppressed separately, we observed that the score had a significant reduction in time (all *p*-values < 0.001). Moreover, we were not able to see a significant differences of AKASI in time (T1 vs. T0 and T2 vs. T0) between the two groups of patients (*p*-value 0.444 and 0.006, respectively). In Table 5 we reported also the percentage reduction of AKASI between time (part A) and we observed that among T0-T2 there was an average percentage reduction in AKASI score of 31.37% for immunocompetent and 22.76% among immunosuppressed patients (part B).

**TABLE 5:** The AKASI reduction in time in both immunocompetent and immunosuppressed patients.

PART A	Period evaluated	Patients evaluated	Difference of mean ± SD
Immunocompetent	T0-T1	39	−0.76 ± 1.33
	T0-T2	37	−1.59 ± 1.58
Immunosuppressed	T0-T1	29	−0.90 ± 1.07
	T0-T2	27	−0.90 ± 1.30

**PART B**	**Period evaluated**	**Patients evaluated**	**Mean percentage changes ± SD**

Immunocompetent	T0-T1	39	−14.11% ± 31.68
	T0-T2	37	−31.37% ± 40.08
Immunosuppressed	T0-T1	29	−25.17% ± 37.12
	T0-T2	27	−22.76% ± 35.37

**FIGURE 3:**
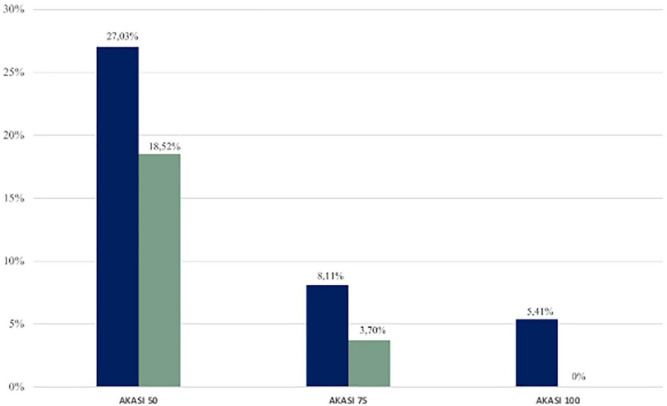
Percentages of immunocompetent (blue) and immunosuppressed (green) patients who achieved AKASI 50, 75, and 100.

Then, we calculated the number (and the relative percentages) of subjects who had an improvement in AKASI scores at the end of the study of 50%, 75%, and 100% (Figure 3).

In the immunocompetent group, the AKASI 100 (complete clearance) was achieved by 2 patients (5.41%), the AKASI 75 by 3 (8.11%), and the AKASI 50 by 10 (27.03%).

In the immunosuppressed group, no patient achieved an AKASI 100, whereas AKASI 75 was reached by 1 patient (3.70%) and AKASI 50 in 5 (18.52%).

**FIGURE 4:**
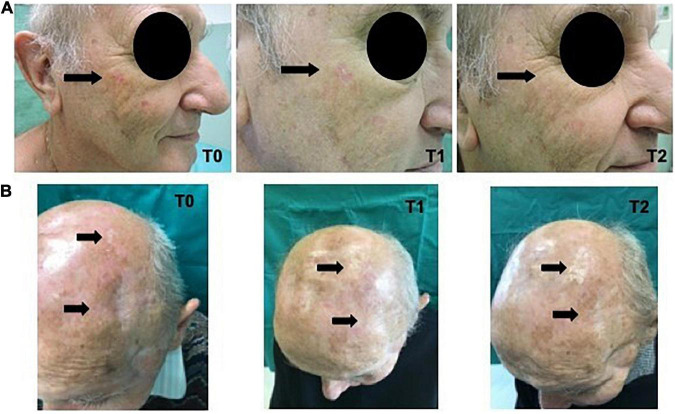
**(A)** Immunosuppressed patient. **(B)** Immunocompetent patient.

In Figure 4 the clinical images of two treated patients, from T0 to T2, are reported.

### NIRS results

Analysis with NIRS spectroscopy was conducted at T0 and T2 on a smaller group of 15 patients (8 immunocompetent and 7 immunosuppressed).

The MANOVA did not show significant differences for the O_2_Hb and HHb signals in the two analyzed epochs (before and after ice application) between pre- and post-treatment (Table 6).

**TABLE 6:** *P*-value and d of the O_2_Hb and HHb signals in the two epochs before and after.

	O_2_Hb before	O_2_Hb after	HHb before	HHb after
***p*** **-value**	0,122	0,169	0,218	0,116
**d**	0	0	0	0

The *p*-value and d (dimension of the space in which the variances of the groups fall) allow us to understand if the two groups show statistical differences and therefore can be considered as two distinct groups (*d* = 1). In our case, the calculated values do not allow a distinction.

**FIGURE 5:**
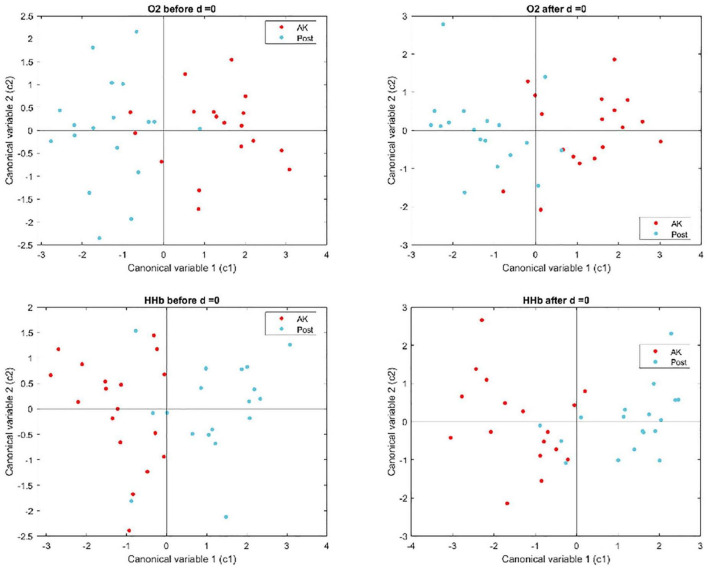
Graphical representation of the first two canonical variables estimated through the MANOVA for the O_2_Hb and HHb signals, before and after ice application.

Figure 5 shows the first two canonical variables estimated through the MANOVA, in the four conditions of the O_2_Hb and HHb signals before and after ice application.

## Discussion

The rising incidence of AKs, in particular in immunosuppressed subjects, and the high risk of evolution of these “*in situ*” neoplasms toward invasive tumors, make their treatment and the identification of appropriate tools for response monitoring essential.

The use of high-protection UV filters has proved to be useful against the skin chronic photoirradiation damage (8, 9). However, recent studies have shown that new medical devices are more effective in primary prevention, especially in higher-risk patients (12–16). In a previous randomized controlled study (17), we demonstrated the effectiveness of a new class I medical device containing active ingredients with antioxidant and repairing action for the prevention and treatment of AKs in a cohort of 90 patients, which also included 28 chronically immunosuppressed subjects. At the end of the treatment, the reduction of the mean number of AKs was 54.7% in the treatment group, vs. 9.43% among the patients who had only used sunscreen. The present study represents the continuation of the previous one, employing the new formulation of the product, characterized by the replacement of vitamin E with nicotinamide. This is a water-soluble derivative of vitamin B3, that demonstrated, both *in vitro* and *in vivo* several photoprotective effects, enhancing DNA repair, reducing the UV-induced suppression of skin immune responses, modulating inflammatory cytokine production, and restoring cellular energy levels after UV exposure (22, 23). In particular, in our experience, this molecule has proven able to protect human primary keratinocytes isolated from the FC from the UV-induced oxidative stress (24). Among the active ingredients of the product tested in this study there are also green tea polyphenols (epigallocatechin gallate) that demonstrated immune protective and antioxidant effects (25) and DNA repairs enzymes. Recent studies confirmed the effectiveness of the topical application of these molecules in the prevention and treatment of actinic keratoses. Notably, the T4 Endonuclease V (T4N5), derived from the UV-resistant bacterium *Micrococcus luteus*, significantly reduced actinic keratoses in treated patients (26, 27). Similar effects were obtained also by the treatment with topical products containing photolyase, derived from marine cyanobacteria and by the combination of high protection traditional sunscreens, endonuclease and photolyase (28, 29). Also, *in vitro* and *in vivo* experiments suggested a putative role in skin cancer prevention of OGG-1, another DNA repair enzyme derived from the mustard plant *Arabidopsis thaliana*, even its effective efficacy has yet to be confirmed in the clinical setting (30).

In this study, the assessment of the clearance of lesions in treated patients was performed using the AKASI score. This method allows to analyze both the extension of the actinic damage affected area, and the clinical characteristics of AK. Compared to the simple count of the lesions, the AKASI score has some advantages: i) it is a reproducible and reliable method; ii) takes greater account of the actinic damage within the FC area and iii) solves the difficulty of lesion counting in patients with severe photodamage, where often the AKs are coalescing within an erythematous and inflamed area (18). Furthermore, the skills for the AKASI calculation are easily acquired and the calculation rapid, and therefore suitable for an assessment in the outpatient setting. In this study, the evaluations were carried out by 4 different dermatologists; nevertheless, the inter-operator reproducibility of the AKASI score has recently been confirmed (31).

In our study, the mean AKASI score calculated at T0 was significantly higher among the immunocompetent patients than in the OTRs (4.47 vs. 3.21; *p*-value 0.013). This discrepancy may be justified by the fact that the mean age, and consequently the risk of chronic actinic damage (32), was significantly higher in the immunocompetent group (77.67 vs. 68.16; *p*-value < 0.001). It should also be noted that the immunocompromised subjects enrolled in this study followed a periodic surveillance program and had been trained in the constant use of photoprotectors, which have been proven to be sufficiently effective in the prevention of actinic keratoses (9–11, 13). Moreover, a prevalence of subjects with phototype II was observed in the OTRs group (88.10% vs. 28.13) with a consequent predominancy of phototypes III in the immunocompetent group. It should be remembered that all the patients enrolled in our study presented phototype II and III. In fact, the phenotypic characteristics of the population hailing from the geographical area in which it was conducted make phototype I very rare; furthermore, a mandatory inclusion criterion was the presence of AKs, that are extremely unfrequent in subjects with brown/black skin (phototypes IV, V, and VI) (32, 33).

A significantly higher AKASI score (*p*-value 0.019) was found in subjects with a previous NMSC history. This data agrees with a retrospective study which analyzed the association between AKASI score and risk of evolution from Aks to iSCC, demonstrating that the patients with AKASI > 7 may have a higher risk of developing iSCC (19).

In our experience, the AKASI score proved to be a valid tool to verify the efficacy of the product under study, highlighting an average percentage reduction at the end of treatment of 31.37% in immunocompetent patients and 22.76% in organ transplant recipients, in comparison to the initial values. The reduction in AKASI values was statistically significant in the single time intervals (T0 vs. T1 and T1 vs. T2) in both groups of treated patients. The demonstration of the efficacy of the treatment also in the group of solid organ recipients represents a particularly important data, in consideration on the high propensity of these subjects to develop skin neoplasms (4), and of the scarcity of literature to support the efficacy and the safety of medical devices for the prevention and treatment of AK and FC in this specific subset of patients (17). In fact, the reduced efficiency in tumor surveillance, resulting from the long-term use of immunosuppressive drugs, could cause a lower capacity to respond to treatments.

In our study, the use of the AKASI score also allowed us to identify treatment response thresholds to evaluate the treatment outcomes, based on those prosed by Schmitz et al. (34), with a complete clearance of the lesions (AKASI 100) reached in 2 immunocompetent patients and the achievement of AKASi 75 in 8.11% of the immunocompetent and 3.7% of the immunosuppressed patients, and of AKASI 50 in 27.03% and 18.52%, respectively.

Another innovative aspect of our project was the use of NIRS, a non-invasive technique that evaluates hemoglobin relative concentration variations, for the assessment of the response to treatment. The feasibility of this method for the AK monitoring has already been demonstrated in previous studies conducted by our group (20, 21), in which we identified through a multivariate analysis of variance a different vascular response in AK compared to healthy skin and in the lesions themselves before and after treatment with Imiquimod 3.75%. In the present study, however, despite the evidence of clinical response to treatment, we did not find significant differences for O_2_Hb and HHb signals in the two periods. At baseline condition (before epochs), the vascularization of the AK lesions before and after the topical cream did not show statistically significant differences. This result confirms those shown in our previous study, in which NIRS signals did not detect any significant differences between the vascularity in AK lesions before and after Imiquimod 3.75% (35). In the previous study, the HHb signals showed statistically significant differences only after ice application, demonstrating the importance of the vasoconstrictor stimulus for the vascular response assessment. Notably, the two groups of signals showed a statistical difference only after the application of the stimulus. In the present study, both HHb and O_2_Hb signals showed no significant differences, even after the application of the vasoconstrictor stimulus. The dissimilarity between the intense pro-inflammatory and immunomodulating effect exerted by imiquimod (35), and the mechanism of action of the product used in the current study could justify this possible incongruity. Its ingredients, in fact, acts by repairing the UV-induced DNA damage and reducing the oxidative stress provoked by the photo exposure, but have only a moderate anti-inflammatory action and do not significantly modify the vascularization of the treated areas (22, 23, 36). The absence of statistical significance could also be due to the relative scarceness of the sample monitored by NIRS, compared to the study previously conducted (21), or to a different severity and extent of the lesions assessed. Unfortunately, the studies are not easily comparable on this point, since the evaluation of the extension was previously carried out in agreement with the grading proposed by Olsen (37) and not through the AKASI score.

## Conclusion

Herein, we demonstrate the effectiveness in AK prevention and treatment of a topical product that combines high-protection sunscreens with anti-oxidant molecules and enzymes capable to repair photo-induced DNA damages, confirming the validity of this therapeutic strategy even in patients under long-term immunosuppressive treatment.

Based on our experience, AKASI has proven to be a valuable tool in monitoring these patients, while further large-scale studies will be needed to confirm the possible application of NIRS in this setting.

Due to the high incidence of chronic actinic damage and the related lesions (*i.e.*, FC, AK, and iSCC) both in the general and chronically immunosuppressed population, we believe that the results deriving from this study may represent strategies of interest for the treatment and monitoring of patients suffering from these pathological conditions.

## Data availability statement

The raw data supporting the conclusions of this article will be made available by the authors, without undue reservation.

## Ethics statement

The studies involving human participants were reviewed and approved by Comitato Etico Interaziendale AOU Maggiore della Carità di Novara. The patients/participants provided their written informed consent to participate in this study.

## Author contributions

FV, SS, KM, EZ, and PS contributed to conception and design of the study. MB organized the database. CA performed the statistical analysis. PS wrote the first draft of the manuscript. FV, SS, KM, CA, and EZ wrote sections of the manuscript. All authors contributed to manuscript revision, read, and approved the submitted version.
